# Supplementary material for: Preparing to act follows Bayesian inference rules
Source: iScience. 2025 May 12;28(6):112645. doi: 10.1016/j.isci.2025.112645 (PMC12159885; doi:10.1016/j.isci.2025.112645)
Supplement: Document S1. Figures S1–S9, Tables S1–S3, and Methods S1–S3 [file mmc1.pdf]

iScience, Volume *28*

Supplemental information

Preparing to act follows Bayesian inference rules

Luca Tarasi, Chiara Tabarelli de Fatis, Margherita Covelli, Giuseppe Ippolito, Alessio Avenanti, and Vincenzo Romei

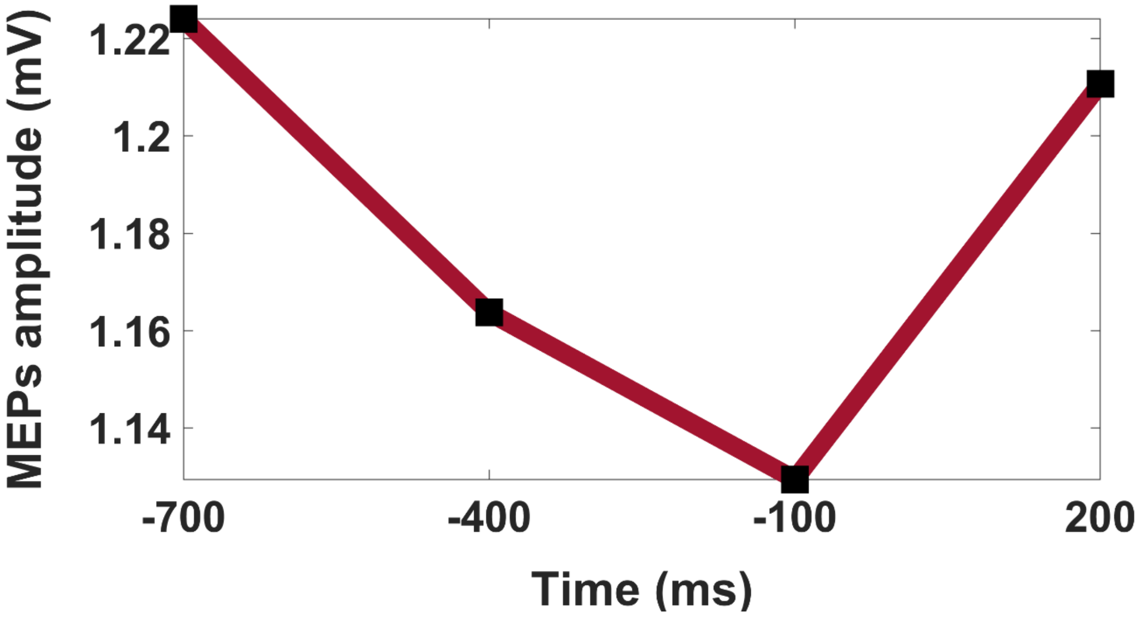

Figure S1: Time effect on MEPs amplitude, related to Figure 2.

As discussed in the main text, we conducted a three-way ANOVA on MEPs amplitude to investigate the influence exerted by prior expectations on motor system excitability. Specifically, we considered prior information (3 levels: right, neutral, left), time (4 levels: -700, -400, -100, +200 ms) and hand (2 levels: right, left) as within factors. We found a significant interaction between hand and prior, indicating different prior-dependent modulations between the two hands. Furthermore, there was a significant effect of TMS timing on MEPs activity ($F_{3,183}$=14.23, p<0.001), but any significant interaction with the other factors considered (i.e., hand and cue), meaning that MEP amplitude changed as a function of time, but the effect was the same regardless of the presented cue or the considered hand. For this reason, we decided to collapse the time factor in subsequent analyses and considered the four TMS conditions (-700ms, -400ms, -100ms, +200ms) altogether. Here we have represented the main factor of time, which demonstrates a progressive decrease in cortical excitability as the stimulus approaches, followed by a rise when the stimulus is presented.

Methods S1: Adopting More Liberal Criteria for MEPs Removal Does Not Affect Results, related to STAR Methods.

Processing of electromyographic (EMG) included the removal of MEPs showing precontractions above 0.3 mV in the preceding 350ms and MEPs below 0.1 mV, which led us to a 12% of trials removed. This widely employed strategy allows for the elimination of trials where it is unclear whether an MEP has been elicited or if the recorded activity is merely due to stochastic fluctuations in electromyographic activity

As a control analysis, we applied more liberal criteria, discarding only MEPs showing precontractions above 0.3 mV in the preceding 350ms before TMS pulse. This procedure led to a 1% of trials removed. We replicated the three-way ANOVA on MEPs amplitude considering the remaining trials. As reported in the STAR Methods section, we considered prior information (3 levels: right, neutral, left), time (4 levels: -700, -400, -100, +200 ms) and hand (2 levels: right, left) as within factors. This control analysis revealed the same pattern of results, showing a significant interaction between hand and prior ($F_{2,122}$=13.79; p<0.001) and a main effect of time ($F_{2,122}$=17.12; p<0.001). Post-hoc analyses indicated the presence of a congruency effect between hand and prior. In the right hand, MEPs were higher vs. lower when the rightward (1.20±0.07) vs. leftward (1.08±0.06, t_{61}=--2.92; p=0.005, BF=6.39) cue was presented. With the neutral prior, MEPs were higher (1.19±0.07) relative to the leftward condition (t_{61}=-3.34; p=0.001; BF=19.23) but not significantly different from the rightward condition (t_{61}=-0.53; p=0.60; BF=0.16).

As for the left hand, higher activity was found when the leftward prior was presented (0.98±0.06) relative to when the rightward prior was presented (0.90±0.06; t_{61}=4.20; p<0.001; BF=238.57). In the neutral condition (0.94±0.06), MEPs were higher than in the rightward condition (t_{61}=3.36; p<0.001; BF=20.14) and lower than in the leftward condition (t_{61}=2.30; p=0.025; BF=1.60). The reported analyses highlight the same pattern of results observed when adopting more conservative criteria for MEPs removal, thus validating the results from our first analyses. Crucially, the same pattern of results emerges when we consider only the three pre-stimulus timings (i.e., -700, -400, -200) in the ANOVA, whether using the conservative or liberal criteria for MEP removal.

Methods S2: Removing the data from the TMS pulse delivered after the stimulus presentation does not affect the results, related to STAR Methods.

Our paradigm consisted of four stimulation conditions, where TMS pulses were delivered at different timings relative to stimulus onset (-700, -400, -100, +200ms). As a control analysis, we investigated MEPs amplitude modulations considering only pre-stimulus conditions, thus removing from data analysis trials in which the TMS pulse was delivered 200ms after stimulus onset. We replicated the three-way ANOVA on MEPs amplitude with prior information (3 levels: right, neutral, left), time (3 levels: -700, -400, -100ms) and hand (2 levels: right, left) as within factors. The ANOVA showed a significant interaction between hand and cue ($F_{2,122}$=12.39; p<0.001) and a main effect of time ($F_{2,122}$=17.36; p<0.001; See Fig.S1 for graphic representation of the temporal evolution in MEPs amplitude). We further explored the interaction hand*cue using paired sample one-tailed t-tests. The right hand showed higher vs. lower MEPs when the rightward (1.30±0.07) vs. leftward cue (1.20±0.06; t_{61}=2.78; p=0.004) was presented. With the neutral cue, MEPs were higher (1.31±0.07) relative to the leftward condition (t_{61}=3.27; p<0.001) but not significantly different from the rightward condition (t_{61}=-0.35; p=0.64). The left hand showed higher vs. lower MEPs when a leftward (1.12±0.06) vs. rightward cue (1.02±0.05; t_{61}=4.16; p<0.001) was presented. The neutral cue led to higher MEPs (1.09±0.06) relative to the rightward cue (t_{61}=3.62; p<0.001) and lower MEPs relative to the leftward cue (t_{61}=1.88; p=0.033). These results corroborate our initial analysis, indicating that the hand-dependent effect of priors on MEP activity described in the main text was not guided by a post-stimulus process.

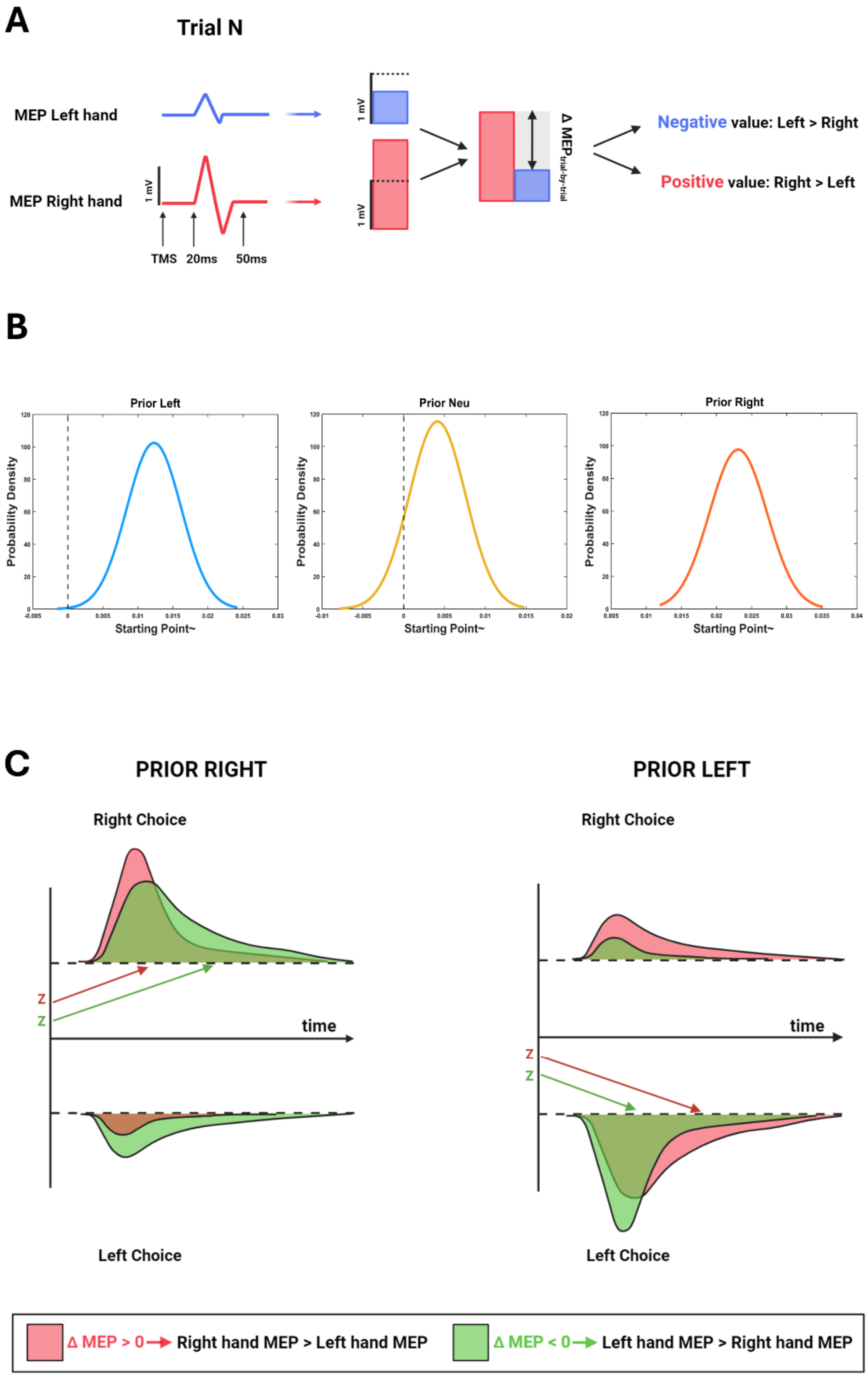

Figure S2: Alternative DDM model confirms original results, related to STAR Methods.

A*. The second DDM model confirmed the results of the first one (see Main text). Indeed, it demonstrated that the model 1 results remained valid even after removing the congruency effect. Specifically, it showed that trials where the MEPs of the right hand were relatively larger in amplitude than those of the left hand were*

associated with a stronger rightward decisional bias. Conversely, when the MEPs of the left hand exceeded those of the right hand, there was a bias towards reporting leftward movement.

***B**. Crucially, this effect interacted with the provided priors. In conditions where the prior was informative (i.e., right and left), the significant effect persisted ($p<0.001$). However, in conditions where the prior was uninformative, MEP fluctuations did not predict the shift in the starting point parameter ($p=0.12$).*

***C**. This suggests that the trial-by-trial effect is not merely a stochastic mechanism, where the decision shifts based on which M1 area exhibits stronger pre-activation. Instead, it demonstrates a prior-driven effect, which intentionally triggers the modulation of MEPs congruent with the prior, subsequently impacting the decision even at the trial-by-trial level.*

	Component Loadings	
	**PC1**	**PC2**
AQ – Social skills	0.712	**-0.360**
AQ – Attention switching	0.525	**-0.438**
AQ – Attention to detail	0.257	0.655
AQ – Imagination	0.192	**-0.391**
AQ – Communication	0.664	-0.090
SPQ – Reference	0.449	**0.369**
SPQ – Magical thinking	0.311	**0.779**
SPQ – Social anxiety	0.644	-0.343
SPQ – Unusual perceptual experiences	0.581	**0.592**
SPQ – Odd behavior	0.672	0.312
SPQ – No close friends	0.759	-0.156
SPQ – Odd speech	0.568	0.253
SPQ – Constricted affect	0.595	-0.271
SPQ – Suspiciousness	0.603	-0.252

Table S1: Loadings of AQ and SPQ questionnaires subscales in PCA components, related to STAR Methods.

Principal component analysis isolated two components that together explain 53% of the variance. The questionnaire loadings are consistent with those identified in the literature, with the first component showing a positive correlation with all subscales, collapsing the common information between the two conditions. The second component, on the other hand, traces the ASD-SSD continuum, having positive loadings with the positive subscales of the SPQ and negative loadings with the subscales of the AQ.

Additional correlation analyses between MEP and Criterion modulation

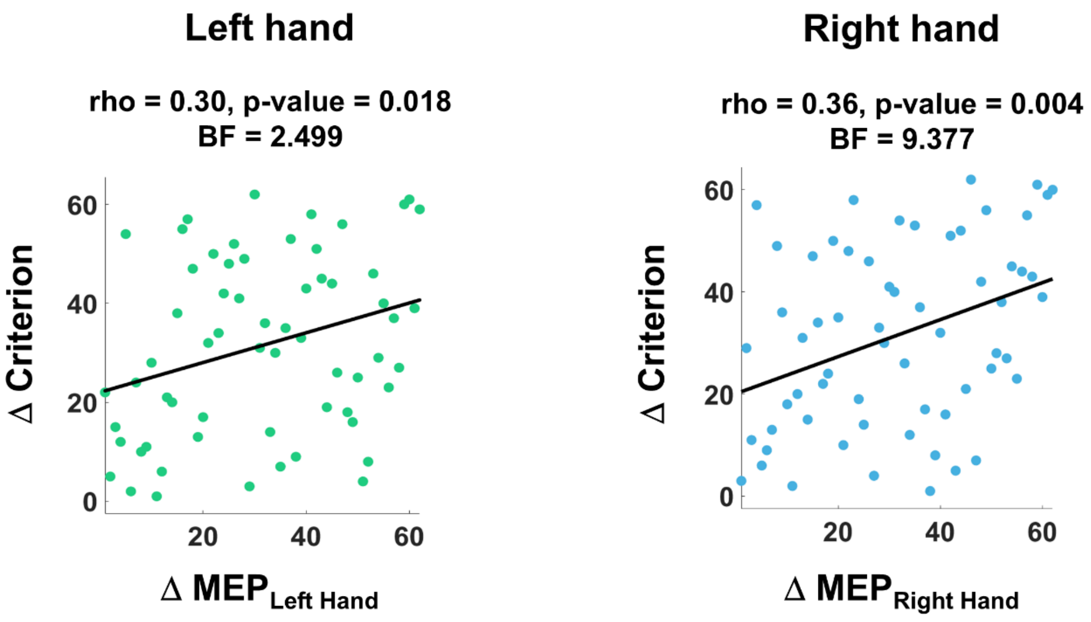

Figure S3: Correlation between criterion and MEP modulations in the left and right hands, related to STAR Methods.

As reported in the main text, we investigated the relationship between prior-related effects on decision behavior and on motor excitability by employing Spearman correlation analysis between ΔCriterion and $\Delta MEP_{trial\text{-}averaged}$. While in the analysis presented in the main text we used a single MEP modulation index for the two hands together ($\Delta MEP_{trial\text{-}averaged} = \Delta MEP_{right\_hand} + \Delta MEP_{left\_hand}$), we conducted additional correlation analyses to evaluate whether the relationship between behavioral and physiological measures held true when considering the right and the left hands separately. ΔMEP for the two hands were computed by subtracting MEP amplitude in the congruent condition to MEP amplitude in the incongruent condition (e.g., $\Delta MEP_{left\_hand} = MEP_{left\_cue} - MEP_{right\_cue}$). Results showed positive correlations between ΔCriterion and both $\Delta MEP_{left\text{-}hand}$ (rho=0.30, p=0.018, BF=2.499) and ΔMEP_{right_hand} (rho=0.36, p=0.004, BF=9.377). In both cases, larger prior-driven criterion modulations lead to larger variation in MEP amplitudes as a function of prior expectations. This provides further support to what we discussed in the main text, showing that motor excitability and decision criterion are strongly related.

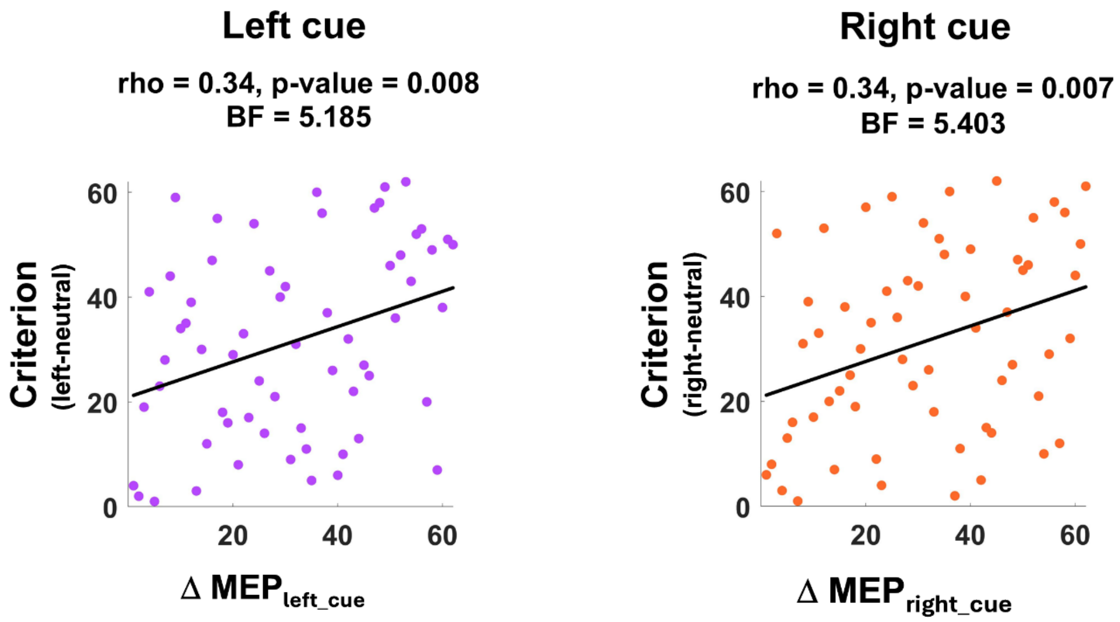

Figure S4: Correlation between criterion and MEP modulations with the left and right cues, related to STAR Methods.

In addition to the correlation analysis reported in the main text, we investigated the relationship between criterion and MEP amplitude separately for each informative cue, by computing ΔMEP as the difference between MEPs in the right and left hand. Instead of using raw MEP values, for each hand we corrected the amplitudes associated with the left and right cues for the neutral condition ($MEPs_{(right\text{-}neutral)}$ and $MEPs_{(left\text{-}neutral)}$). The correction for the uninformative condition, in which no directional expectancies were involved, was applied due to significant differences in CSE levels between hands, in order to allow a better comparison of CSE modulations. Then, we calculated two deltas based on the corrected MEP amplitudes: ΔMEP_{left_cue} (right $MEPs_{(left\text{-}neutral)}$ – left $MEPs_{(left\text{-}neutral)}$) and ΔMEP_{right_cue} (right $MEPs_{(right\text{-}neutral)}$ – left $MEPs_{(right\text{-}neutral)}$). Positive ΔMEP values indicate that right MEPs are higher than left MEPs, while negative ΔMEP values indicate that left MEPs are higher. For criterion modulation, we applied a similar correction approach by calculating two prior-specific values corrected for the neutral condition ($criterion_{(leftt\text{-}neutral)}$ and $criterion_{(right\text{-}neutral)}$).

For the left cue, ΔMEP exhibited negative values (-0.16±0.04), indicating that corrected MEP amplitudes were greater in the left relative to the right hand, whereas for the right cue ΔMEP showed positive values (0.06±0.03), indicating the opposite trend. In contrast, criterion values were positive in the left cue condition (0.50±0.08) and negative in the right cue condition. To facilitate interpretation of the relationship between ΔMEP and

criterion, we inverted the sign of criterion values. This transformation ensured that both ΔMEP and criterion values were negative with the left cue and positive with the right cue.

Spearman correlation analyses were conducted between prior-specific ΔMEP and Criterion, showing a significant positive relationship in both conditions (Left cue: rho=0.34, p=0.008, BF=5.185; Right cue: rho=0.34, p=0.007, BF=5.403). This means that the greater the modulation of MEPs between the prior-congruent and prior-incongruent hand, the stronger the directional bias.

Methods S3: TMS timing control (ANOVA on d' and c)

To explore potential effects on sensitivity and response criterion related to TMS-timing, two ANOVAs were conducted on d' and c including both cue type (3 levels: righ, neutral, left) and TMS timing (4 levels: -700, -400, -100, +200 ms) as within factors. For both d' and c, the ANOVAs showed significant interactions between Timing and Cue (d': $F_{3, 366}=3.45$, $p=0.003$; c: $F_{3, 366}=3.23$, $p=0.004$).

Specifically, regarding sensitivity, d' values with the right cue in the +200ms condition are lower relative to all the other conditions (all $t_{61} \geq 3.89$, all $p<0.001$; for statistics see Table S1), indicating a drop in participants' sensitivity when the TMS pulse follows stimulus onset. All the other comparisons were not significant (see Table S1). For the left cue, the only significant difference was between the -700ms and -100ms condition ($t_{61}=3.05$, $p=0.003$), while the comparison between the +200 and the -700 condition was marginally significant ($t_{61}=1.95$, $p=0.055$). No other comparison reached significance (see Table S1). Finally, with the neutral cue no significant differences were found across conditions (see Table S1).

Concerning response criterion, post-hoc analyses revealed that in the +200ms condition values were higher with the left cue (all $t_{61} \leq -2.30$, all $p \leq 0.025$; statistics are reported in Table S2) and lower with the right cue (all $t_{61} \geq 2.10$, all $p \leq 0.040$) compared to the other conditions, with the only exception of the comparison between +200 and -100 with the right cue ($t_{61}=1.11$, $p=0.272$). All the other comparisons did not reach statistical significance either with the left cue or the right cue (see Table S2). As observed for sensitivity, no modulations emerged to be significant with the neutral cue (see Table S2).

To summarize, results showed that criterion displays in general more extreme values (i.e., higher with the left cue and lower with the right cue) when the TMS pulse is delivered after stimulus onset, when evidence accumulation for decision-making is already taking place. On the other hand, at least regarding trials with the right cue, sensitivity seem to decrease in that same condition, while remaining stable during left cue trials. Furthermore, results on the neutral condition revealed no modulation in either criterion or sensitivity, indicating that the effect of TMS timing depends specifically on the type of cue received and cannot be attributed to a non-specific influence of TMS pulses on behavioral measures.

The large influence of the +200ms condition on these results, as demonstrated by the fact that nearly all significant comparisons regard this condition, suggests that, in contrast with analyses on MEPs (see the paragraph "Removing the data from the TMS pulse delivered after the stimulus presentation does not affect the results" where the ANOVA on MEP amplitudes excluding the +200ms condition was excluded), criterion

values are modulated when the stimulation occurs after stimulus appearance. Specifically, in the +200 condition the presentation of the left (or the right) cue leads to a stronger bias toward left (or right) responses compared to the other conditions.

Table S2: TMS-timing x Cue effect on sensitivity (d'), related to STAR Methods.

**SDT sensitivity (d')**						
**T-tests** (df=61)	*t*			*p-value*		
	Left cue	*Right cue*	*Neutral cue*	*Left cue*	*Right cue*	*Neutral cue*
-700 vs +200	1.95	3.89	1.38	0.055	**<0.001**	0.174
-400 vs +200	0.83	4.59	0.71	0.412	**<0.001**	0.483
-100 vs +200	-0.88	4.26	0.08	0.385	**<0.001**	0.940
-700 vs -400	1.42	-1.50	0.61	0.161	0.139	0.543
-700 vs -100	3.05	-0.87	1.61	**0.003**	0.386	0.113
-400 vs -100	1.71	0.56	0.65	0.092	0.578	0.515

**Statistics**			
**Condition**	mean±SEM		
	Left cue	*Right cue*	*Neutral cue*
-700	1.17±0.08	1.02±0.08	1.16±0.07
-400	1.05±0.07	1.16±0.09	1.10±0.09
-100	0.90±0.06	1.11±0.08	1.04±0.07
+200	0.98±0.08	0.73±0.07	1.03±0.08

Table S3: TMS-timing x Cue effect on criterion (c), related to STAR Methods.

**SDT criterion (c)**						
**T-tests** (df=61)	*t*			*p-value*		
	Left cue	*Right cue*	*Neutral cue*	*Left cue*	*Right cue*	*Neutral cue*
-700 vs +200	-2.35	2.62	-0.30	**0.022**	**0.011**	0.763
-400 vs +200	-2.30	2.10	-1.01	**0.025**	**0.040**	0.316
-100 vs +200	-2.69	1.11	-1.27	**0.009**	0.272	0.209
-700 vs -400	-0.47	0.34	0.68	0.640	0.734	0.498
-700 vs -100	0.30	1.78	1.03	0.764	0.081	0.309
-400 vs -100	0.78	1.15	0.33	0.436	0.256	0.743

**Statistics**			
**Condition**	mean±SEM		
	Left cue	*Right cue*	*Neutral cue*
-700	0.37±0.07	-0.39±0.08	-0.08±0.05
-400	0.39±0.09	-0.40±0.08	-0.11±0.06
-100	0.36±0.08	-0.46±0.08	-0.13±0.05
+200	0.49±0.08	-0.51±0.08	-0.07±0.05

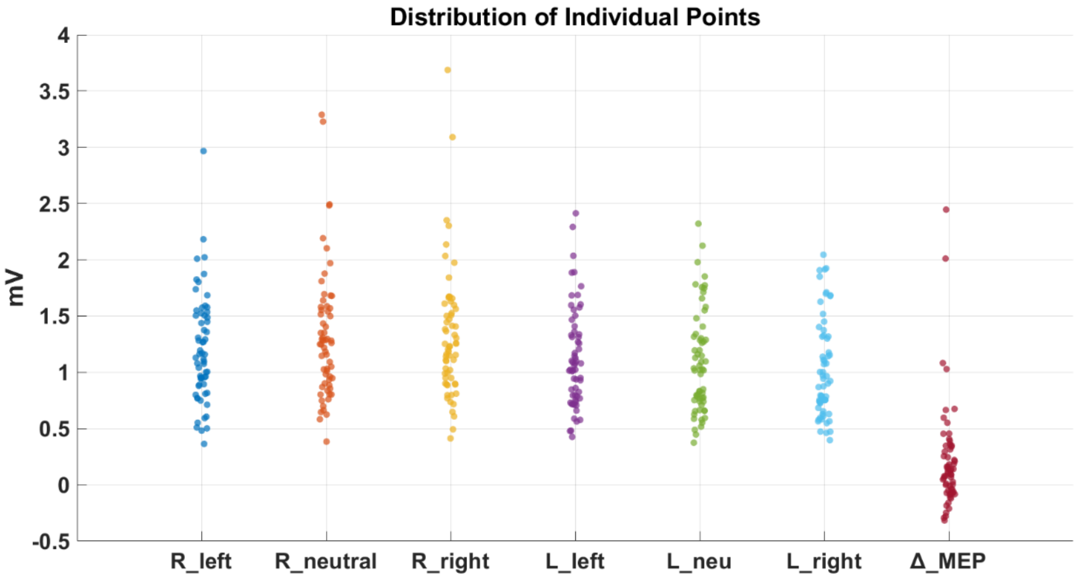

Variable		Median	IQR	Shapiro-Wilk (W)	p-value
**Right hand**	Left cue	1.15	0.61	0.96	**0.025**
	Neutral cue	1.26	0.66	0.90	**<0.001**
	Right cue	1.23	0.61	0.88	**<0.001**
**Left hand**	Left cue	1.07	0.57	0.95	**0.015**
	Neutral cue	1.02	0.57	0.95	**0.009**
	Right cue	0.93	0.59	0.93	**0.001**
**$\Delta MEP_{trial\text{-}averaged}$**		0.11	0.38	0.72	**<0.001**

Figure S5: Distribution of individual MEPs, related to STAR Methods.

Representation of individual data relative to mean MEP amplitudes in the left and right hands under the three cue conditions and individual $\Delta MEP_{trial\text{-}averaged}$. *Statistics of distributions and results from Shapiro-Wilk tests are reported in the table above. Shapiro-Wilk tests revealed that the distribution of all variables is non-normal (all* $p<0.05$*). Therefore, as reported in the main text, post-hoc analyses on MEP amplitudes comparing different conditions were conducted employing Wilcoxon-signed rank tests, while Spearman correlations were used to explore the relationship between MEPs and decision-making behavior. Furthermore, the results reported in the main text using raw MEP amplitudes were replicated using log-transformed data (See the paragraph "Analyses on log-transformed MEP data confirms results on raw MEP data").*

Methods S4: Analyses on log-transformed MEP data confirms results on raw MEP data, related to STAR Methods.

As mentioned in the main text (see also Fig. S6), as MEP amplitudes were not normally distributed, we applied a log-transformation to the data and repeated the analyses. Results showed the same pattern as the ANOVA on raw MEP data. Specifically, we found a significant interaction between Hand and Cue ($F_{2, 122}$=13.73, p<0.001), and a significant main effect of TMS timing ($F_{3, 183}$=15.35, p<0.001). Post-hoc comparisons relative to the hand*cue interaction highlighted the same pattern from the original analysis: for the right hand, log-MEPs were lower after left cue (0.69±0.02) relative to both the right (0.74±0.03, t_{61}=-2.84, p=0.006) and neutral cues (0.73±0.03, t61=-3.50, p<0.001), without any significant difference between the right and neutral conditions (t_{61}=-0.27, p=0.788). Similarly, for the left hand log-MEPs were lower after the right cue (0.62±0.02) relative to both the left (0.66±0.02, t61=4.23, p<0.001) and neutral cues (0.65±0.02, t_{61}=3.26, p=0.002). In addition, log-MEPs were lower in the neutral relative to the left condition (t_{61}=2.30, p=0.025).

Furthermore, we repeated correlation analyses between prior-related MEP and criterion modulations, this time using log-transformed MEP values to compute ∆MEPtrial-averaged. Spearman correlation showed a positive relationship between ∆MEPtrial-averaged and ∆criterion (rho=0.38, p=0.003, BF=13.653), indicating that a greater influence of prior expectations on decisional criteria is associated with a greater modulation of motor system excitability.

Correlation analyses between MEP and Starting Point modulation

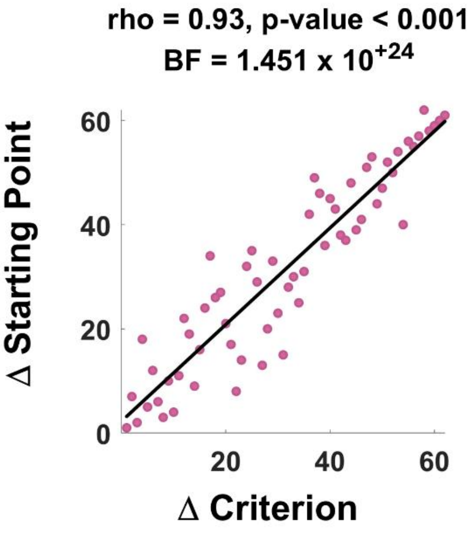

Figure S6: Correlation between criterion and Starting Point modulations, related to STAR Methods.

We conducted a single-subject level analysis of the Drift Diffusion Model (DDM), focusing on the starting point (z), a parameter reflecting decisional bias analogous to the criterion in Signal Detection Theory (SDT), making it highly relevant to our study. Our analysis confirmed that z and criterion, though distinct parameters, depict the same bias-related process, as indicated by a strong positive correlation between changes in z (∆z: z(left) - z(right)) and Criterion (∆Criterion: c(left) - c(right); rho=0.93, p<0.001, BF=1.451x10^{+24}). Furthermore, we explored the relationship between z-values and corticospinal excitability (CSE) modulations. Given the strong correlation between z and criterion values, a significant relationship between z and EMG activity was expected.

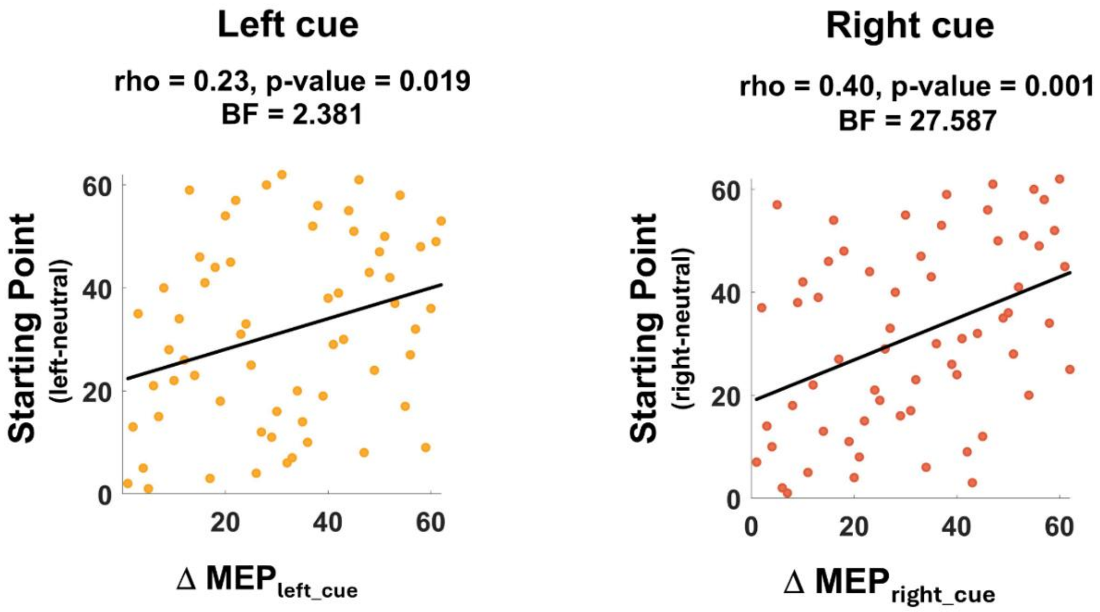

Figure S7: Correlations between Starting Point and MEP modulations for the left and right cues, related to STAR Methods.

To deepen our understanding of the relationship between z-values and MEP modulations, we conducted separate correlation analyses for left and right cues, controlling for baseline response biases by subtracting values obtained under the neutral condition. This approach mirrored the procedure previously applied in criterion analyses (see reviewer 1, point 4). Specifically, we examined the correlations between z(left-neutral)/z(right-neutral) and ΔMEPcue left/ΔMEPcue right, respectively. Consistent with prior findings on bias modulation reflected through the SDT criterion parameter, we observed significant positive correlations for both right cues (rho=0.40, p=0.001, BF=27.587) and left cues (rho=0.23, p=0.019, BF=2.381). These results further corroborate the relationship between prior-related effects on motor excitability and the diffusion decision model (DDM) z-values. Overall, these findings provide strong evidence that decisional biases, as indicated by shifts in the z, are closely linked to motor excitability as measured through MEPs, reinforcing the view that biases reflected in DDM parameters have a measurable impact on neural activity, further connecting cognitive decision-making processes with motor system dynamics. These results reinforce the importance of cortical excitability as a mediating factor in understanding how placement along the SSD-ASD continuum influences decisional bias.

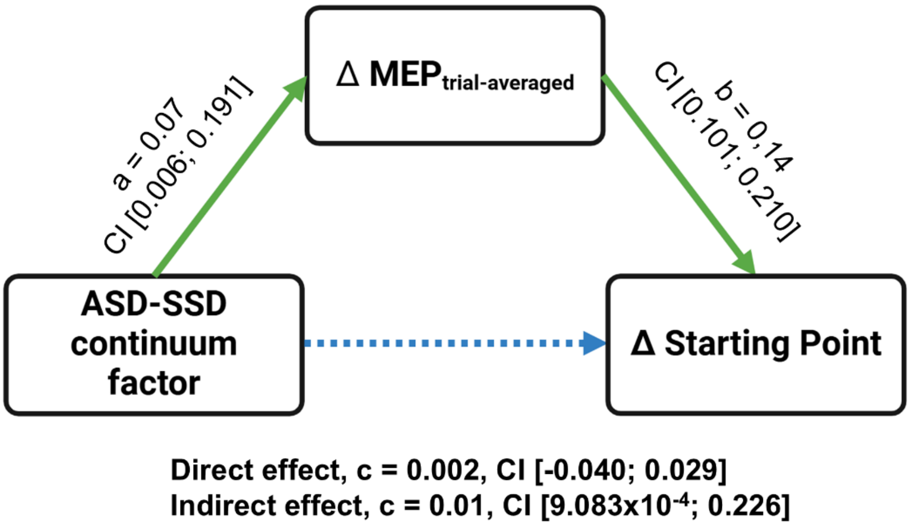

Figure S8*:* Mediation with delta-z*,* related to STAR Methods.

To further validate the findings of our initial mediation analysis, where we observed that changes in motor excitability mediated the relationship between PC2 and SDT criterion, we conducted a second mediation analysis with Δz, a measure of decisional bias derived from the Drift Diffusion Model (DDM), as the dependent variable. This analysis aimed to examine whether the observed mediating effect of motor excitability on decisional bias (as indicated by shifts in the decision criterion) was consistent when z-values were used as the outcome. The results were consistent with our earlier findings. Specifically, we observed an indirect effect of PC2 on Δz (indirect effect: c = 0.01, CI [9.083x10^{-4}; 0.226]), indicating that the relationship between PC2 and decisional bias was mediated by modulations of motor excitability. However, no direct effect of PC2 on Δz was found (direct effect: c = 0.002, CI [-0.040; 0.029]), further confirming that the impact of PC2 on decisional bias was fully mediated through motor excitability.

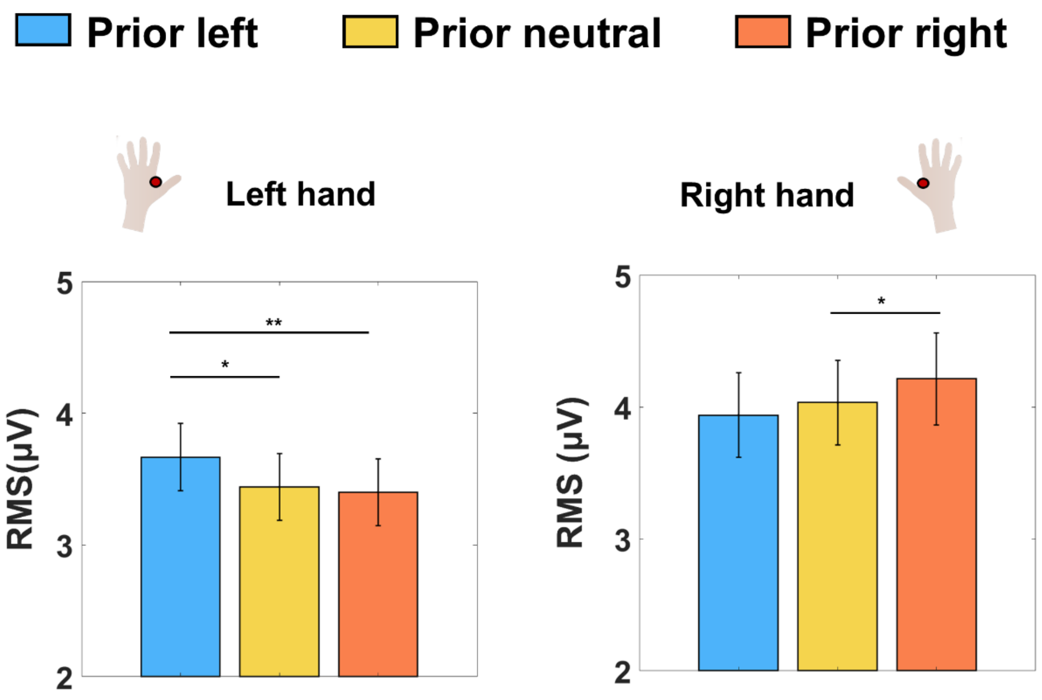

Figure S9: RMS values under the three cue conditions, related to STAR Methods

In order to ensure that observed effects on MEPs are not confounded by changes in muscle activity, we extracted pre-pulse EMG root mean square (RMS) values for each condition, performing similar analyses to those conducted on MEPs.

The ANOVA conducted on pre-pulse RMS values revealed a significant interaction between Hand and Cue ($F_{2,122}=4.85$, $p=0.009$), indicating that cue-related modulations of those values are different between the two hands. Given the non-normality of the distributions, we further explored this effect through Wilcoxon signed-ranked tests. Results showed that, in the right hand, higher RMS values were observed with the right cue (4.21µV±0.35) relative to the neutral cue (4.03µV±0.32; $z=-2.24$, $p=0.015$), but not to the left cue (3.94µV±0.32; $z=-1.84$, $p=0.066$). The comparison between left and neutral cue was not significant as well ($z=-0.49$, $p=0.625$). As for the left hand, RMS values were higher with the left cue (3.67µV ±0.26) relative to both the neutral (3.44µV ±0.25; $z=2.26$, $p=0.024$) and the right cues (3.40µV ±0.25; $z=3.06$, $p=0.002$), while no significant difference emerged between neutral and right cues ($z=1.26$, $p=0.210$).

Mean RMS values in µV are represented in the figure below (bars represent standard error) separately for the left and right hands as a function of prior.

These results partially align with the ANOVA on MEPs amplitude. For the left hand, MEPs were higher with the left cue relative to the others. In contrast, while MEPs differed between right and neutral cues, RMS values remained stable across those two conditions. For the right hand, an opposite pattern emerged relative to MEP analyses. While RMS values differed only between right and neutral cues, significant differences in MEP amplitudes were found between left and both neutral and right cues.

We also conducted Spearman correlation analyses between RMS and response criterion modulations. Following the approach applied on MEP data, we computed ∆RMS as the difference of RMS values in congruent vs. incongruent conditions for each hand separately (e.g.,∆RMS_right hand = RMS with the right cue – RMS with the left cue) and then summed those two values to obtain a general index for cue-related RMS modulation. We then investigated whether this modulation correlated with behavioral effects (∆criterion = criterion with the right cue – criterion with left cue). Results revealed a significant positive correlation between ∆RMS and ∆criterion (rho=0.27, p=0.031, BF=1.52), indicating that stronger effects of cues on decision-making behavior are associated with higher modulations of RMS values.

However, this association emerged to be considerably weaker than the one between MEPs and criterion (rho=0.41, p=0.001, BF=32.54). Moreover, when replicating the analysis on left and right hand separately, a significant correlation emerges only for the right hand (rho=0.30, p=0.018, BF=2.44) but not for the left hand (rho=0.18, p=0.171, BF=0.40), while both resulted to be significant when using MEP values (right hand: rho=0.36, p=0.004, BF=9.38; left hand: rho=0.30, p=0.018, BF=2.50). Again, even when considering the significant association between criterion and RMS in the right hand, correlation indices and BF factors are weaker relative to the association of behavior with MEP amplitude.

Furthermore, to assess whether differences in RMS values across conditions could have driven the effects on MEP amplitudes, we replicated correlation analyses between ∆criterion and ∆MEP but controlling for ∆RMS. Results showed that, even when accounting for RMS modulations, the association is still significant (rho=0.32, p=0.011). In contrast, correlation between RMS and criterion when controlling for ∆MEP does not reach the significance threshold (rho=0.08, p=0.56).

We further investigated the role of MEPs and RMS on decision behavior through a generalized linear mixed model using logistic regression. Specifically, we calculated the difference between MEP values in the congruent vs. incongruent hand on a trial-by-trial basis (e.g., in trials preceded by the left cue: ∆MEP=MEP in the left hand – MEP in the right hand) and normalized these values using z-score transformation. We replicated

the same procedure with RMS values (e.g., ∆RMS=RMS in the left hand – RMS in the right hand). Higher values in ∆MEP (or ∆RMS) indicate that MEP (or RMS) in the congruent hand was higher than MEP (or RMS) in the incongruent hand. We then assessed whether these indices drove participants' choices, with higher values in either ∆MEP or ∆RMS associated with stronger tendency toward prior-congruent responses.

Trials were defined as "congruent" or "incongruent" based on the alignment of participants' responses with prior information (e.g., a trial is congruent if presentation of the left cue is followed by a left response). As participants tend to give prior-congruent responses, leading to a higher number of congruent trials, we calculated inverse proportional weights based on congruency. These weights were further scaled by the size of the larger congruency class to ensure balanced contributions of both congruent and incongruent trials to the statistical model.

Specifically, trial weights for each participant were computed to be inversely proportional to the number of trials in the corresponding category (i.e., for congruent trials: w = 1/number of congruent trials; for incongruent trials: w = 1/number of incongruent trials). Weights were then adjusted by multiplying them for the size of the largest congruency class. This way, higher weight was assigned to incongruent responses (i.e., less frequent responses) than congruent responses, and balanced contribution of both congruency classes to the regression model was ensured.

A generalized linear mixed model was fitted using weighted data to investigate whether ∆MEP and ∆RMS predict response congruency. To account for within-subject variability, a random intercept was included for each participant. Logistic regression was performed in Matlab using logit function and binomial distribution. This analysis revealed that only MEP modulation predicted the congruency of participants' responses (β=0.098, SE=0.016, t=6.241, df=12548, $p<0.001$) while the effect of RMS was not significant (β=0.004, SE=0.015, t=0.228, df=12548, p=0.820).

In summary, these results highlight how the observed effect on MEPs and decision bias cannot be fully attributed to RMS changes, corroborating the interpretation provided in the main text.
